# 3D printing polylactic acid polymer-bioactive glass loaded with bone cement for bone defect in weight-bearing area

**DOI:** 10.3389/fbioe.2022.947521

**Published:** 2022-07-25

**Authors:** Yurun Ding, Xiaolin Liu, Jue Zhang, Zhuocheng Lv, Xiangchao Meng, Zhiguo Yuan, Teng Long, You Wang

**Affiliations:** Department of Bone and Joint Surgery, Renji Hospital, School of Medicine, Shanghai Jiaotong University, Shanghai, China

**Keywords:** bone cement, bioactive glass, 3D printing, bone regeneartion, polylactic acid

## Abstract

The treatment of bone defects in weight-bearing areas is mainly to transplant filling materials into the defect area, to provide immediate and strong support for weight-bearing. At present, the commonly used filling material is bone cement, which can only provide physical support without bone regeneration effect. The long-term stress at the interface may cause the loosening of bone cement. The ideal filling material should provide not only strong mechanical support but also promote bone regeneration. We introduce a 3D printing frame-filling structure in this study. The structure was printed with polylactic acid/bioactive glass as the frame, and bone cement as the filler. In this system, bone cement was used to provide immediate fixation, and the frame provided long-term fixation by promoting osteogenic induction and conduction between the interface. The results showed that the degradation of bioactive glass in the frame promoted osteogenic metabolism, induced M2 polarization of macrophages, and inhibited local inflammatory response. The *in vivo* study revealed that implantation of the frame-filling structure significantly promoted bone regeneration in the femoral bone defect area of New Zealand white rabbits. For a bone defect in a weight-bearing area, long-term stability could be obtained by bone integration through this frame-filling structure.

## 1 Introduction

Bone defect in the weight-bearing area is difficult to treat clinically. The treatment requires providing instant and stable support for the defect site, which can enable the affected limb to carry out the weight-bearing exercise as soon as possible. Currently, the main treatment is to place filling materials at the defect site to provide mechanical support for the weight-bearing area (Stahl and Yang, 2021). These materials include autologous bone, allogeneic bone, and bone cement. Polymethylmethacrylate (PMMA) is commonly applied to bone cement clinically, which is used to fill local bone defects such as joint replacement and percutaneous vertebroplasty (Laende et al., 2021; Prost et al., 2021). PMMA has high mechanical strength after curing and can provide sufficient initial mechanical support. However, due to its inert characteristic, PMMA is not easy to degrade and does not have bone regeneration bioactivity. After filling the bone defect in the weight-bearing area with bone cement alone, long-term weight-bearing may produce fatigue damage to bone cement, resulting in the failure of fixation.

For a bone defect in the weight-bearing area, the ideal treatment needs to restore weight-bearing function immediately, and more importantly, to promote new bone formation effectively for long-term biological stability. The filling material needs to provide strong initial mechanical support and show degradability and bone regeneration ability. Moreover, the filling material should display plasticity to meet the morphological diversity of bone defects. Some studies modified bone cement by adding bioactive agents, such as calcium, phosphorus, or bone morphogenesis protein, in order to increase bone induction activity (Castro-Raucci et al., 2018; Ruskin et al., 2020). However, the mixture reduced the compressive strength of bone cement and could not meet the initial stable fixation of bone defects in the weight-bearing area. Moreover, the active components were wrapped in bone cement and could not effectively exert their bioactivity.

Polylactic acid (PLA) is a polyester polymer material polymerized with lactic acid as the main raw material, which has good biocompatibility and degradability. PLA has been widely used in clinics, such as absorbable anchors for ligament or meniscus fixation, absorbable sutures, vascular stents, and other implant materials (Matsuki et al., 2018; Singhvi et al., 2019; Zhang et al., 2019). PLA has a low melting point and good plasticity. However, the disadvantage of PLA is its low biological activity, which needs to be modified to improve its performance. Bioactive glass (BG) is a silicate glass composed of SiO_2_, Na_2_O, CaO, and P_2_O_5_. It has good biocompatibility, bioactivity, and degradability. The trace elements released during the degradation, such as calcium, silicon, phosphorus, magnesium, and strontium, can exchange ions with body fluids, form a hydroxyapatite (HA) layer similar to the natural bone on the material surface, and then form a solid chemical bond with the adjacent bone surface, which can promote the formation of new bone. In addition, silicon and calcium released by BG can up-regulate osteoblast activity at the gene level to promote osteogenesis (El-Rashidy et al., 2017; Zheng et al., 2021). However, BG itself lacks reliable plasticity and low mechanical strength, which is difficult to meet the biomechanical requirements of bone defects in weight-bearing areas.

According to the requirements of mechanical support and bone induction for bone defects in weight-bearing areas, we designed a frame-filling structure with PLA, BG, and bone cement. We blended PLA with BG nanomicrospheres, and then constructed a PLA-BG framework structure by 3D printing additive manufacturing technology. 3D printing technology can prepare scaffolds according to the shape of bone defects, and the preparation process does not change the physical and chemical properties of the raw materials. The frame structure not only has the plasticity of PLA but also has the bone-induced bioactivity of BG. The degradability of the frame structure provides space for bone growth. PMMA bone cement, as the filler of the frame-filling structure, ensures the initial mechanical requirements for the bone defect in the load-bearing area.

## 2 Materials and methods

### 2.1 Fabrication of 3D-printed scaffold using PLA-BG filaments

PLA-BG filaments (diameter: 400 µm) were prepared according to a previous study (Distler et al., 2020). Composite filaments were produced using PLA as the bulk matrix material and BG as a filler. 45S5 BG (composition: 45 wt% SiO_2_−24.5 wt% CaO−24.5 wt% Na_2_O−6 wt% P_2_O_5_, d50 (4.0 ± 1.0) µm, d95: ≤20μm, Schott VitryxxR, Schott AG, Germany) was used. A PLA powder was selected (PLA RXP 7503, Resinex GmbH, Germany).

Cubical scaffolds (length: 10 mm, width: 10 mm, height: 1 mm) were designed with an interconnected porosity and pore diameter of 400 µm using computer-aided design software solid edge (Siemens AG, Germany) and the browser-based CAD tool, Tinkercad (Autodesk Inc., United States ). PLA-BG filaments with 1 and 5% (wt) BG content were fed into a fused deposition modeling (FDM) 3D printer (Ultimaker S5 Premium, Ultimaker B.V., Netherlands), and scaffolds were produced.

### 2.2 Characterization of PLA-BG with different concentrations of BG

The composite scaffold PLA-BG containing 1% and 5% BG was prepared by the aforementioned method. The samples were divided into 3 groups: PLA group (PLA scaffold), 1% PBG group (PLA +1% BG composite scaffold), and 5% PBG group (PLA +5% BG composite scaffold).

#### 2.2.1 Scanning electron microscopy

The scaffolds were frozen in liquid nitrogen and fractured with a scalpel to expose the interior. The scaffolds were examined from the top. Scaffolds were sputter-coated with gold for 40 s and imaged (SEM, 15 kV, Hitachi s-4800).

The formation of bone-like phosphorite on the surface was also observed by SEM. Briefly, the samples were immersed in SBF solution for 3 days, then frozen in liquid nitrogen and fractured with a scalpel to expose the interior. Scaffolds were sputter-coated with gold for the 40s and imaged (SEM, 15kV, Hitachi s-4800).

#### 2.2.2 Fourier transform infrared spectrometer

The chemistry of the scaffolds was measured by FTIR (Nicolet Magna 550, Thermo-Nicolet, Madison, WI). Spectra were recorded in the ATR (attenuated total internal reflectance) mode using a Split Pea accessory (Harrick Scientific Corp., Ossining, NY) featuring a 200-µm Si internal reflection element. All the scans were recorded at a resolution of 4 cm^−1^. OMNIC software (Nicolet) was used for data acquisition and spectrum processing.

#### 2.2.3 X-ray photoelectron spectrometer

The surface elemental composition of the scaffolds was assessed using XPS (Eden Prairie, MN). The photoelectrons emitted from the surface of the samples under X-ray excitation were collected at a take-off angle of 45° and were analyzed with a hemispheric electron energy analyzer operating at a pass energy of 187.9 eV. During the analysis, the base chamber pressure was in the order of 1,010 Torr.

#### 2.2.4 Inductively coupled plasma mass spectrometer

The samples were soaked in the buffer solution (PBS) at the ratio of 0.1 g/ml, and placed on the shaking table at 37 °C for 1, 3, and 7 days 0.5 ml of solutions were collected after 1 day, 3 days, and 7 days, and kept in the refrigerator at 4°C. After dilution, the ion concentration of the extracted solution was tested by ICP-MS to obtain the ion concentration of the final leaching solution.

### 2.3 Biocompatibility of PLA-BG *in vitro*

#### 2.3.1 Cell culture

SD rats from the laboratory animal research center of Renji Hospital Affiliated with Shanghai Jiaotong University School of Medicine were used in this study. The experimental protocol was approved by the Animal Ethical Committee of Renji Hospital Affiliated with Shanghai Jiaotong University School of Medicine. All surgical procedures were performed under general anesthesia with an intraperitoneal injection of 1% pentobarbital.

Bone marrow mesenchymal stem cells (BMSCs) from SD rats were obtained as previously described (Yu et al., 2021). SD rats (age: 2 weeks) were sacrificed by dislocating the cervical spine. Tibias and femurs were immediately dissected under aseptic circumstances. Osteoepiphysis were removed, then marrow tissues were dispersed through repeated flushing. The dispersed tissues were forcefully passed through a 19-gage needle to obtain a single-cell suspension. The cells were cultured in a-MEM (Invitrogen, Carlsbad, CA, United States ) supplemented with 10% FBS, 1% penicillin, and streptomycin. The cell suspensions were seeded in 10-cm tissue culturing dishes in a humidified atmosphere containing 5% CO_2_ at 37°C. The medium was changed every 2–3 days to remove nonadherent cells, and adherent cells were passaged until they get confluent. BMSCs were passaged after digestion with 0.25% trypsin/1 mM EDTA. BMSCs at third passage were used for the experiments.

The scaffolds were placed in 48-well tissue-culture polystyrene plates for cell culture experiments. Wells without scaffolds were used as controls. The scaffolds were put into polystyrene 96-well plates (non-tissue culture treated) for cell culture experiments. Scaffolds were sterilized with ethylene oxide (Anderson Products) and degassed for 2 d under a house vacuum.

#### 2.3.2 Immunofluorescence staining of cells

The polarization of macrophages was evaluated by immunofluorescence staining of RAW cells (RAW264.7). PLA, 1%, and 5% BG-PLA extracts were immersed within DMEM respectively for 1d, the leaching solutions were collected. RAW cells were co-cultured with leaching solutions from PLA, 1% and 5% BG-PLA extracts respectively, then fixed with 3.7% formaldehyde (mass/volume in PBS buffer) for 15 min, washed in PBS, and permeabilized with 0.2% by mass Triton X-100 for 5 min. The cells were labeled with iNOS rabbit polyclonal antibody (PA3-030A, ThermoFisher) at 1:250 dilution in 0.1% BSA and incubated for 3 h s at room temperature, and then labeled with goat anti-rabbit IgG secondary antibody, Alexa Fluor 488 conjugate (green, A27034, ThermoFisher) at a dilution of 1:2000 for 45 m at room temperature. The cells were labeled with CD206 polyclonal antibody (ThermoFisher) using a dilution of 1:200 (1 h, 37°C), followed by goat anti-rabbit IgG Alexa Fluor 594 (Red, ThermoFisher). Nuclei were stained with DAPI (Blue, S36938, ThermoFisher).

#### 2.3.3 Confocal microscopy

Confocal microscopy (Nikon A1R) was used to measure cell morphology on PLA, 1% and 5% BG-PLA scaffolds. High-resolution images were captured for nuclei (Blue, DAPI, Beyotime) and actin (Red, 5 μg/ml, FITC-Phalloidin, Cytoskeleton). DAPI staining of nuclei was used to make sure that cell morphology was assessed for single cells. FITC-phalloidin stained actin images were used to assess cell morphology.

#### 2.3.4 Osteogenic differentiation analysis

Osteogenic differentiation of BMSCs was measured by Alkaline Phosphatase (ALP) assay, alizarin red staining, and Sirius red staining.

For ALP assay, PLA, 1%, and 5% BG-PLA extracts were immersed within DMEM respectively for 7 days. Supernatants from each group (*n* = 5) were co-cultured with BMSCs for 7 and 14 days. Cells were extracted into an assay buffer containing 50 mM Tris-HCl, 0.1% Triton-X-100, and 0.9% NaCl (pH 7.6), and the lysate was frozen at −70°C. The lysate samples were thawed, and enzyme activity was analyzed using 0.1 mM 4-p-nitrophenylphosphate as a substrate in an assay buffer containing 0.1 M Tris and 1 mM MgCl_2_ (pH 10.0, Sigma-Aldrich). After incubation at room temperature for 30min, the reaction was stopped by the addition of 0.1 M NaOH, and the absorbance was measured at 405 nm with a plate reader (Spectramax plus384, United States ). Five parallel samples were measured in triplicate. The protein content was determined by Bio-Rad Protein Assay (Bio-Rad Laboratories) with bovine serum albumin as standard. The specific ALP activity was calculated as absorbance at 405 nm/protein mg/ml. For ALP histochemical staining (*n* = 4), the cells were washed with PBS, fixed with citrate-acetone formaldehyde fixative, washed with deionized water, and stained for enzyme activity with the alkaline solution containing naphthol AS-BI phosphate and fast red violet LB base according to the manufacturer’s instruction (alkaline phosphate kit, Sigma-Aldrich).

For alizarin red staining, BMSCs were washed two times with PBS followed by fixation in 96% ethanol for 15 min at room temperature. 1% of Alizarin red solution (Sigma-Aldrich) was added to the fixed cells and incubated for 60 min at room temperature with gentle rotation. Finally, cells were carefully washed three times with PBS and dried. Pictures were taken with an optical microscope (DFC295, Leica) at ×20 magnification.

For Sirius red staining, cells were washed two times with PBS followed by fixation in 96% ethanol for 15 min at room temperature, then incubated in 0.1% Sirius Red solution (Sigma-Aldrich) for 60 min, washing twice in PBS, dehydrating in 100% ethanol, and then clearing in xylene. Pictures were taken with an optical microscope (DFC295, Leica) at ×20 magnification.

#### 2.3.5 Cell viability test

Cell viability was assessed by the CCK-8 test. BMSCs were plated at a density of 5 × 10^3^ cells/well in a 96-well plate in a 0.1 ml culture medium. The cells were cultured for 24 h and then starved for 24 h in a serum-free DMEM medium. Then the cells were co-cultured with supernatants from each group (*n* = 5) for 1, 3, and 7 days at 37°C. On each day, the culture medium and supernatants were replaced and the cells were counted using CCK-8 (Dojindo, Kyushu Island, Japan). Briefly, 10 µL of the kit reagent was added to each well and the cells were incubated for 2 h. Cell viability was determined by measuring the absorbance at 450 and 655 nm with a microplate reader (Microplate Reader 680; Bio-Rad Laboratories, Hercules, CA, United States ). Each experimental condition was analyzed in five wells.

#### 2.3.6 RT-PCR

The difference in osteogenic differentiation ability in gene expression level was examined by quantitative RT-PCR. After BMSCs were co-cultured with leaching solutions from PLA, 1% and 5% BG-PLA extracts respectively, the mRNA expression of OPN, OCN, ALP, and RUNX2 were examined for 3, 7, and 14 days. Total RNA was isolated using TRIzol reagent (Invitrogen), according to the manufacturer’s standard instructions. For reverse transcription of mRNA, random-primed cDNA was synthesized from 2 mg of total RNA using a PrimeScript RT reagent kit (TaKaRa, Dalian, China). Real-time PCR was performed using 2 µL of cDNA product in a 25 µL reaction volume with a 7,500 Real-Time PCR System (Applied Biosystems, Singapore). SYBR Premix Ex Taq II (Takara Biotechnology), specific primers, and 2 µL of cDNA were used in each PCR reaction (95°C for 30 s, 40 cycles of denaturation at 94°C for 5 s, annealing, and extension at 60°C for 30 s). Sense and antisense primers were designed with Primer Express 5.0 based on published cDNA sequences. GAPDH was used as an internal control gene. All real-time PCR reactions were performed in triplicate, and results after calibration with GAPDH expression were calculated using the ^△△^CT method and are presented as fold increase, relative to non-stimulated control

### 2.4 Characteristics and biocompatibility of PLA-BG/BC

#### 2.4.1 Preparation of PLA-BG/BC

The silica gel mold was prepared through 3D printing mold pouring. The mold had a hole of 1 cm wide, 1 cm long, and 2 mm high, which was fit for the printing stent. The bone cement was prepared after the printing stent was placed. As soon as the viscosity of BC met the requirements, 100 mg of BC was added to each stent and extruded into the gap structure of the stent. The samples were obtained after drying.

The samples were divided into 4 groups: PLA group (PLA polylactic acid scaffold), PBG group (PLA +1% BG composite scaffold), PLA-BC group (PLA + PMMA bone cement composite scaffold), and PBG-BC group (PLA +1% BG + PMMA bone cement composite scaffold).

#### 2.4.2 Scaffold characteristic test

The micromorphology of the scaffolds was detected by SEM. The chemistry of the scaffolds was measured by FTIR. The surface elemental composition of the scaffolds was assessed using XPS. The degradation of the complex was detected by ICP. The specific methods were mentioned earlier.

PLA, PBG, PLA/BC, and PBG/BC were respectively soaked in Tris HCl for 6 months. PH values and sample weight changes were detected for all the samples each week for 6 months.

#### 2.4.3 Cell experiments of PLA-BG/BC

Cytotoxicity and Cell viability tests were conducted using CCK-8 and MTT assay. Cell morphology was measured by confocal microscopy. The polarization of macrophages was evaluated by immunofluorescence staining of RAW cells. Osteogenic differentiation of BMSCs was measured by ALP assay and RT-PCR. The specific methods were mentioned earlier.

### 2.5 *In vivo* experiment

3D printed composite scaffolds were implanted into the femoral tunnel of New Zealand rabbits to detect the short-term and long-term fixation effects of various implants (BC, PLA, PBG, PLA-BC, and PBG-BC). The operation process and animal feeding were carried out in Shanghai Jiagan Biotechnology Co., Ltd., with the approval of its ethics committee. The animal experiment was strictly in accordance with the relevant regulations and provisions on the protection of experimental animals formulated by it.

#### 2.5.1 Surgical procedure and scaffold implantation

Thirty New Zealand white rabbits (aged 8–12 weeks and average weight of 3 kg) were randomly divided into 5 groups (BC group, PLA group, PBG group, PLA-BC group, and PBG-BC group), with 6 rabbits in each group. Each rabbit were implanted a sample scaffold in the femoral trochanter of the right hind limb, and the materials were randomly labeled.

Before surgery, samples (BC, PLA, PBG, PLA-BC, and PBG-BC, 5 mm × 5 mm×1 mm) were sterilized by *γ* Radiation. The rabbits were injected with 1% Pentobarbital Sodium (pentobarbital, 80 mg/kg) intravenously. Local disinfection was carried out after skin preparation of the right hind limb. A 1–2 cm longitudinal incision was conducted at the trochanter, exposing the femoral trochanter, and peeling off the periosteum. Using a 1 mm wide bone chisel, a transverse slotting was made at the tuberosity, with a depth of 5–6 mm and the length of 5–6 mm. Then the sample was implanted into the slot so that the lateral edge of the sample was submerged into the femoral trochanter by about 0.5 mm. The wound was closed layer by layer. Penicillin (800000 units/day) was given intravenously 1–3 days after the operation to prevent infection. The rabbits were sacrificed at 4 and 12 weeks after the operation, and the femoral were removed for general observation, then the specimens were fixed with 10% formalin solution for the following analysis.

#### 2.5.2 Micro-CT

The femur samples were photographed by X-ray and then scanned by micro-CT (sky scan 1,076, aartselaar, Belgium). The parameters were 100 kV and 80 μA. The resolution is 18 µm. The rotation angle of scanning was set to 360°. The sky scan TM CT analyzer software was used for three-dimensional reconstruction and quantitative analysis. The parameters included: bone volume fraction (BV/TV), bone area volume ratio (BS/BV), number of trabeculae (TB.N), and trabecular thickness (TB.Th).

### 2.6 Statistical analysis

Data were presented as mean ± standard deviation (SD). Two-tailed analysis of variance (ANOVA) was used for statistical analysis. The differences were considered significant when *p* < 0.05.

## 3 Results

### 3.1 % PLA-BG bad better properties and biocompatibility

**FIGURE 1:**
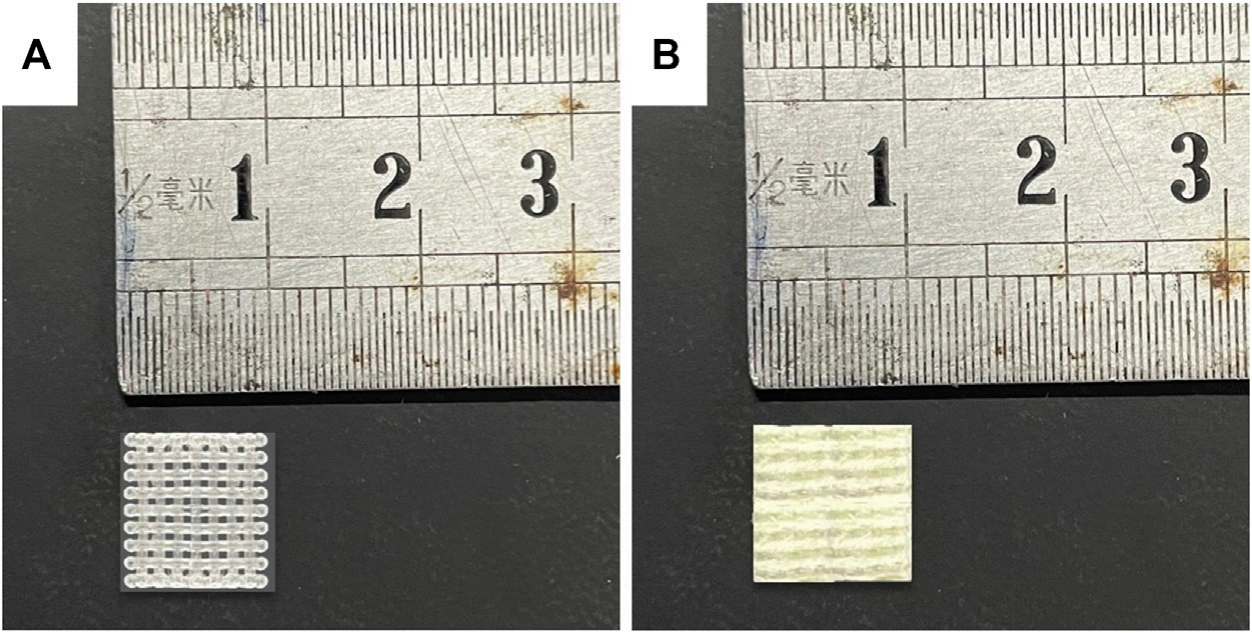
Photos of samples. **(A)** PBG; **(B)** PBG-BC.

The scaffolds were successfully prepared by the FDM method (Figure 1). The *in vitro* results showed that 1% BG-PLA had good biocompatibility, and could promote adhesion, proliferation, and differentiation of BMSCs. 1% BG-PLA showed no toxicity, and the degradation environment of which was neutral. Moreover, it could promote the polarization of macrophages toward M2, and inhibit its polarization towards M1, which is conducive to tissue repair. Therefore, 1% BG-PLA was chosen for the next experiments.

#### 3.1.1 Both 1% and 5% PLA-BG had an amorphous structure

**FIGURE 2:**
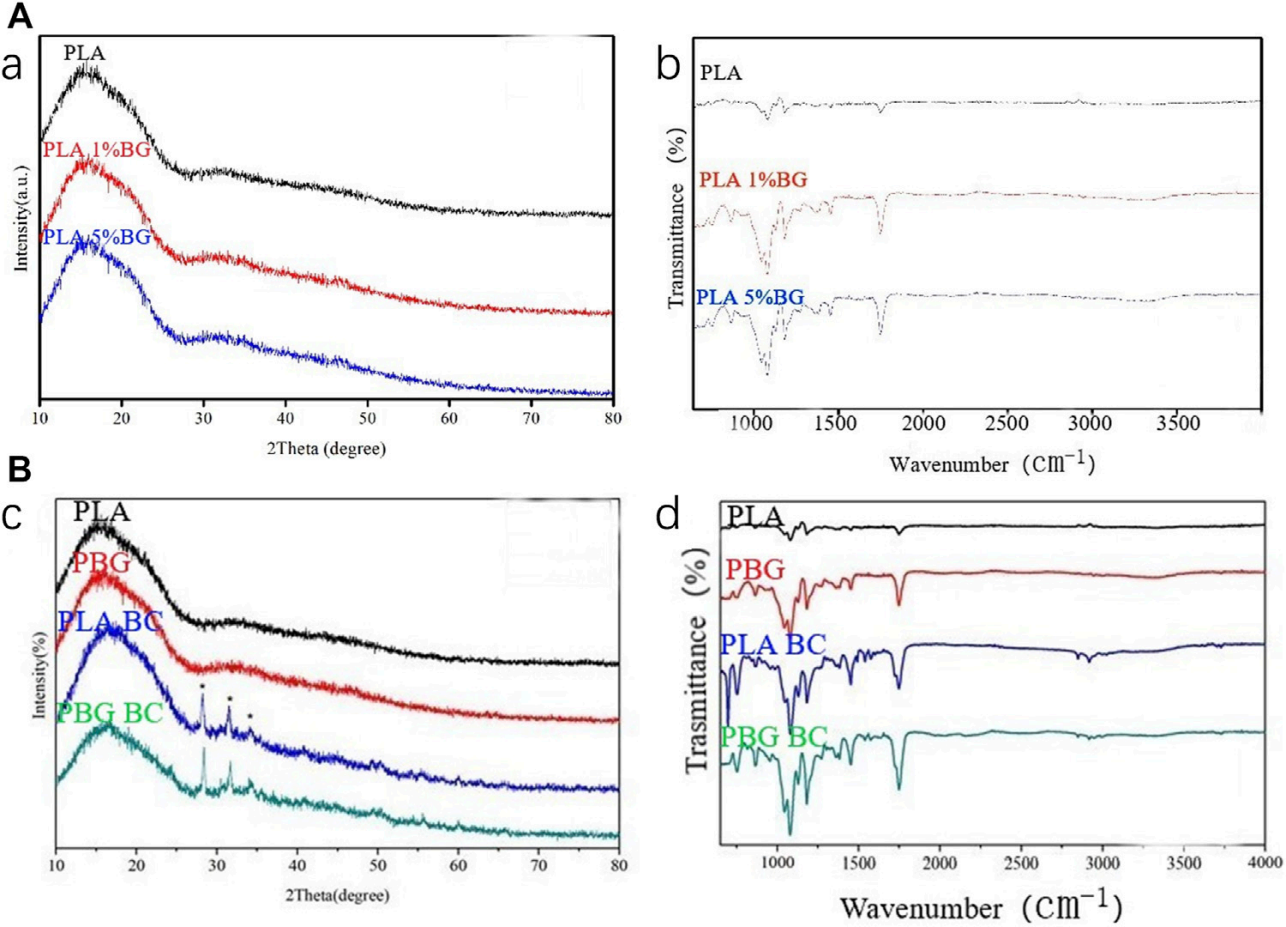
**(A)** XPS and IR spectra of PLA, 1% and 5% BG-PLA; **(B)** XPS and IR spectra of PLA, PBG, PLA-BC, and PBG-BC.

XPS and FTIR were performed to identify the characteristics of PLA, 1% and 5% BG-PLA (Figure 2). The results of XPS revealed that the 3 groups showed obvious steamed bread peaks, indicating the amorphous structure prepared by the 3D method. After adding BG, the waveform did not change significantly, because BG is also amorphous material. The results of FTIR showed peaks at 1,650, 1,250, and 1,130 cm^−1^, which are the characteristic peaks of PLA. After adding BG, there were stretching vibration peaks of Si-O and Si-O-Si around 950 and 1,100 cm^−1^. While no significant difference was observed between 1% and 5% BG-PLA, which might be due to the small content of BG that participated in the energy reaction of the infrared spectrum.

#### 3.1.2 The surface of PLA-BG was rough and presented some micron granular substances. 5% PLA-BG showed more BG particles on the surface

**FIGURE 3:**
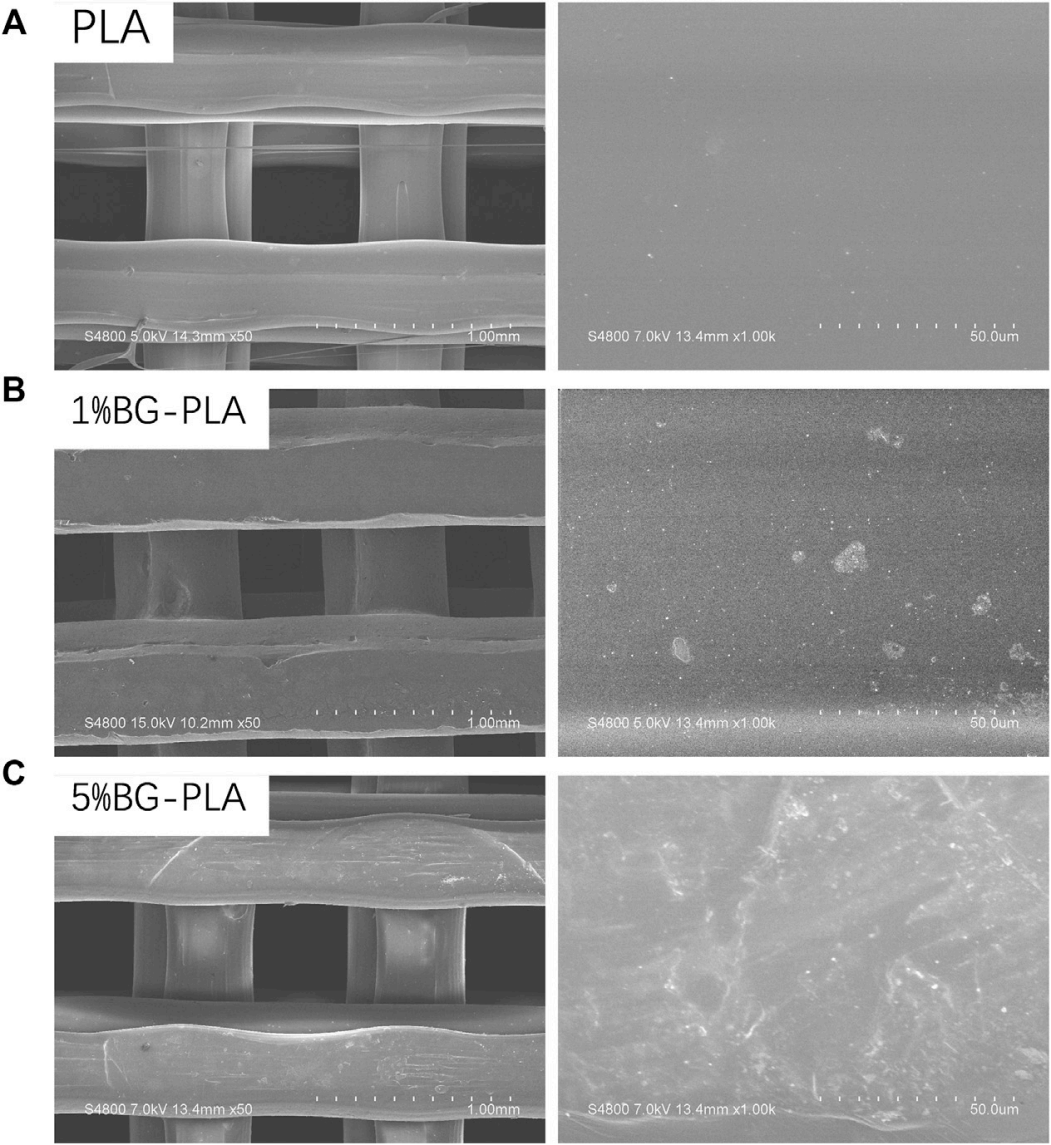
SEM of scaffolds. **(A)** PLA; **(B)** PLA with 1% BG (1%BG-PLA); **(C)** PLA with 5% BG (5% BG-PLA).

The surfaces of three different 3D printing scaffolds were observed by SEM (Figure 3). The diameter of the scaffold fiber was 400 microns, and the gap was also 400 microns. Further amplification revealed that the surface of PLA was relatively smooth. After adding BG, the surfaces became rough and presented some micron granular substances, corresponding to the doped BG particles. The amount of BG particles on the scaffold surface with 5% content was significantly more than that with 1% BG content.

#### 3.1.3 Bone-like phosphorite were formed on the surface of PLA-BG. Ion exchange was more intense on the surface of 5% PLA-BG

**FIGURE 4:**
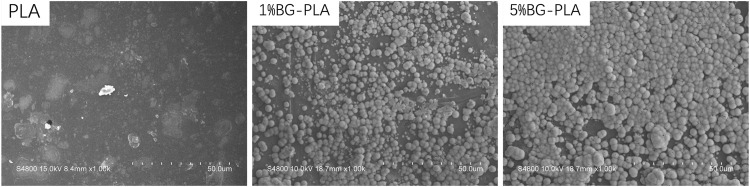
SEM of PLA, 1% and 5% BG-PLA after soaking in SBF solution for 3 days.

After the samples were immersed in SBF solution for 3 days, the formation of bone-like phosphorite on the surface was observed by SEM (Figure 4). Barely any changes were found on the surface of PLA, which was still relatively smooth. Mineralized sediments were found on the surface of 1% and 5% BG-PLA samples, which were typically bone-like phosphorite morphology. This might be due to the fact that BG can induce the formation of surface mineralization by ion exchange and silicon hydroxyl. The amount and distribution of bone-like phosphorite on the 1% surface were less than that on the 5% sample surface, indicating that the ion exchange on the 5% sample surface was more intense, and more mineralized deposits were formed.

#### 3.1.4 PLA-BG could promote M2 polarization of macrophages

**FIGURE 5:**
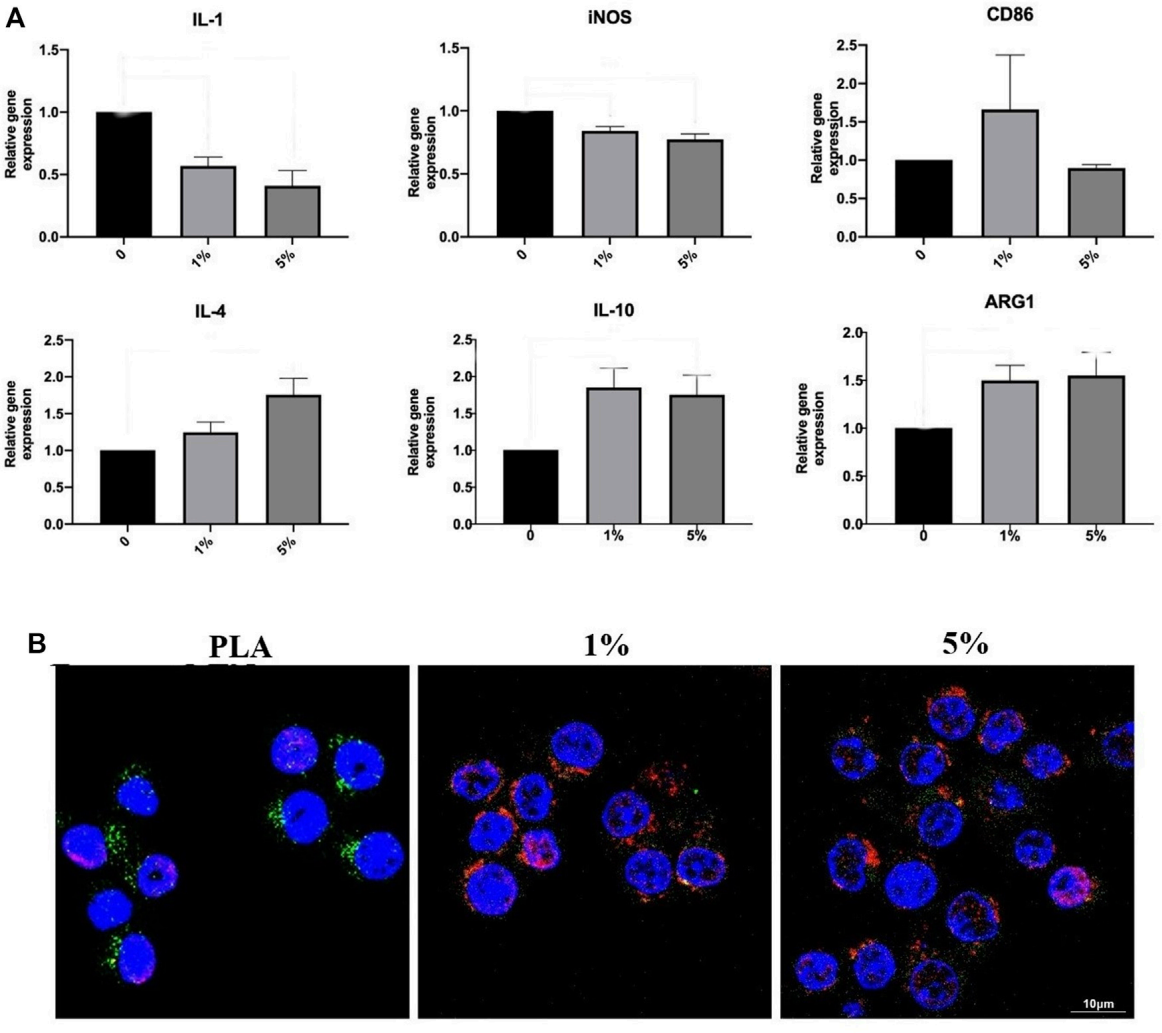
Inducing effect on macrophage polarization of cells RAW264 cultured with leaching solutions from PLA, 1% and 5% BG-PLA. **(A)**. RT-PCR; **(B)** Immunofluorescence.

RT-PCR and immunofluorescence staining were used to evaluate the inducing effect on macrophage polarization of the scaffolds (Figure 5). The results of PCR showed that PLA-BG significantly inhibited the expression of IL-1 and iNOS, while promoting the expression of ARG1 and IL-10. It was suggested that the addition of BG was conducive to the M2 polarization of macrophages. For immunofluorescence staining, green fluorescence represented iNOS and red fluorescence was CD206. The results showed that the degradation or dissolution products of PLA were conducive to the polarization of macrophages towards M1. While after BG was added, the dissolution or degradation products were conducive to the polarization of macrophages in the M2 direction.

#### 3.1.5 1% PLA-BG showed a better proliferation effect

**FIGURE 6:**
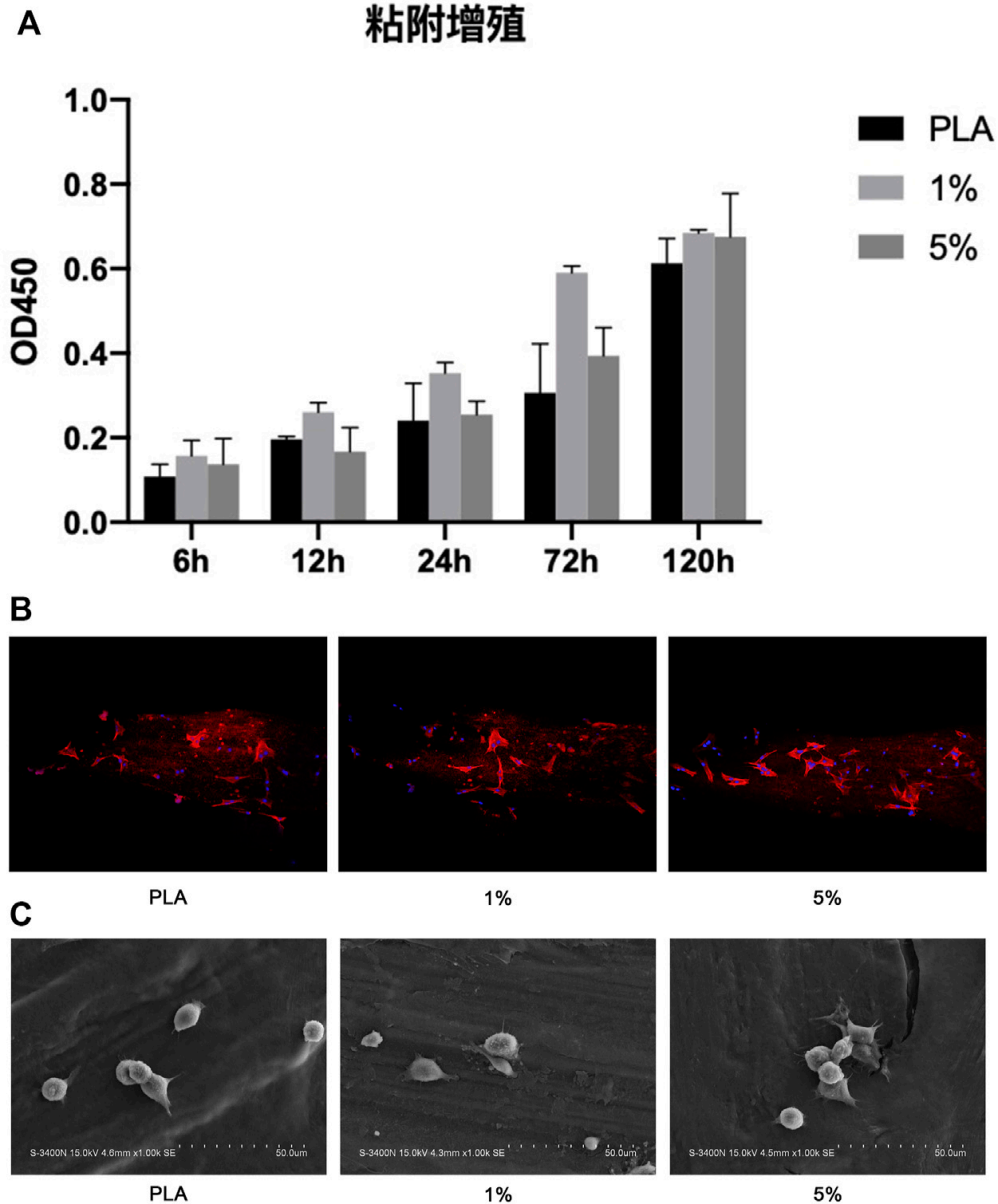
Coculture of BMSCs with scaffolds. **(A)** CCK-8 experiment; **(B)** 3D CLSM pictures of cells on scaffolds (24 h); **(C)** SEM images of cells adhered on scaffolds (12 h).

Cell proliferation was evaluated by the CCK-8 experiment (Figure 6). The results revealed that 1% PLA-BG had a significant promoting effect on cell proliferation during full-time period. While 5% BG-PLA did not show a better promoting effect compared with 1% PLA-BG, even the inhibition of cell proliferation was detected relative to PLA at some time points such as 12 h.

#### 3.1.6 1% and 5% PLA-BG both had better adhesion morphology with more pseudopodia

BMSCs were cultured on PLA scaffolds, 1% and 5% BG-PLA scaffolds for 12 or 24 h. Cell adhesion was observed using CLSM (24 h) and SEM (12 h), respectively (Figure 6). After a coculture of 12 h, the cells began to attach to the sample surface, cells on both 5% and 1% BG-PLA scaffolds had more pseudopodia extending out and attached more rapidly. After coculture of 24 h, the cells exhibited typical spindle morphology. Both 1% and 5% BG-PLA had better adhesion morphology with more pseudopodia.

#### 3.1.7 BG-PLA showed osteogenic differentiation ability

**FIGURE 7:**
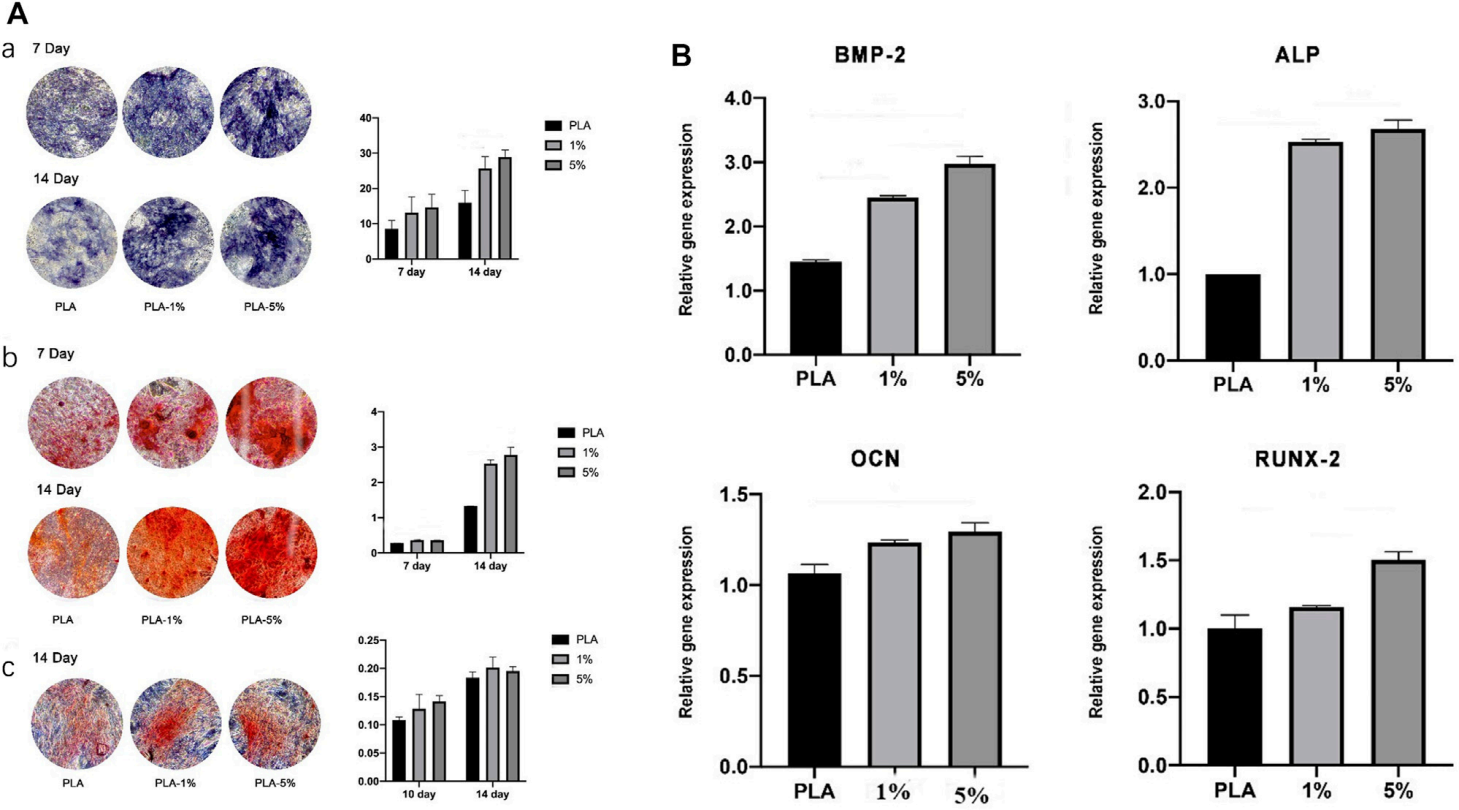
**(A)**. Evaluation of osteogenic differentiation of hBMSCs cultured with leaching solutions from PLA, 1% and 5% BG-PLA. a: ALP staining and statistical analysis; b: Alizarin red staining and statistical analysis; c: Sirius red staining and statistical analysis. **(B)**. Relative expression of osteogenesis-related genes.

The osteogenic activity was evaluated by ALP staining, Alizarin red staining, and Sirius red staining (Figure 7). In 1% and 5% PLA-BG groups, more ALP expression was found by ALP staining, more calcium nodule deposition was found *via* Alizarin red staining, and more collagen formation was found by Sirius red staining. Statistical analysis showed that the positive dyeing areas in 1% and 5% PLA-BG groups were significantly larger than that of the PLA group.

RT-PCR was performed to evaluate the relative expression of osteogenesis-related genes (Figure 7). In 1% and 5% BG-PLA groups, an increased expression of osteoblast markers was shown in comparison to that of the PLA group, indicating that BG induced osteogenic activity.

### 3.2 PBG-BC provided sufficient initial mechanical strength, good biocompatibility, and effective osteogenic induction

The addition of BC provided the initial mechanical strength, without affecting the biocompatibility and osteogenic induction of PBG.

#### 3.2.1 Addition of BC maintained the amorphous structure of PBG

The composition of the PBG-BC surface was analyzed by XPS and FTIR (Figure 2). The characteristic peak of PBG and PMMA was found after BC was filled into PBG, indicating the completion of composite scaffolds containing PMMA and changes in the characteristics of the original PBG.

#### 3.2.2 BC was completely filled into the gap of PBG

**FIGURE 8:**
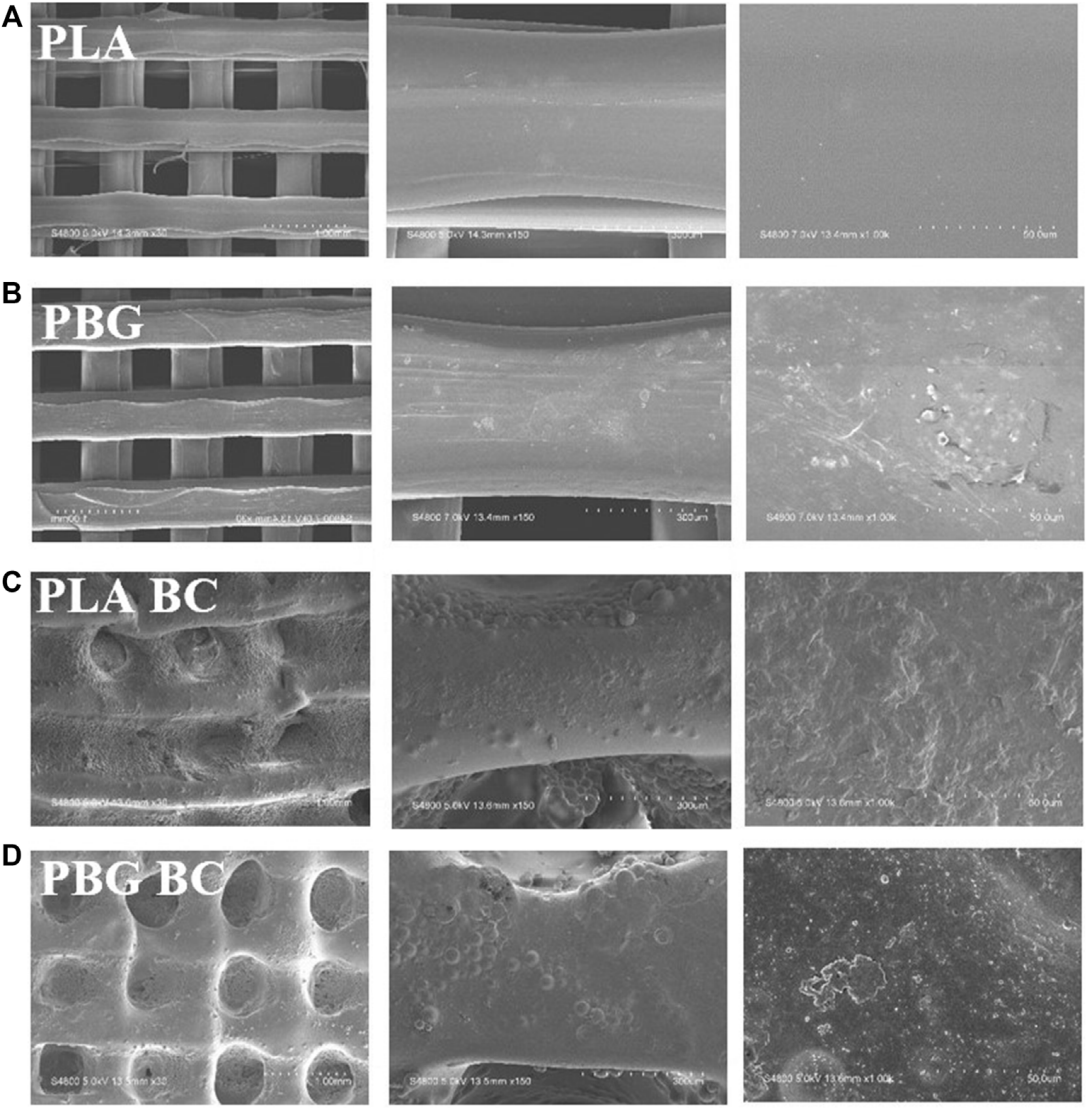
SEM of the scaffolds. **(A)**: PLA; **(B)** PBG; **(C)** PLA-BC; **(D)** PBG-BC.

**FIGURE 9:**
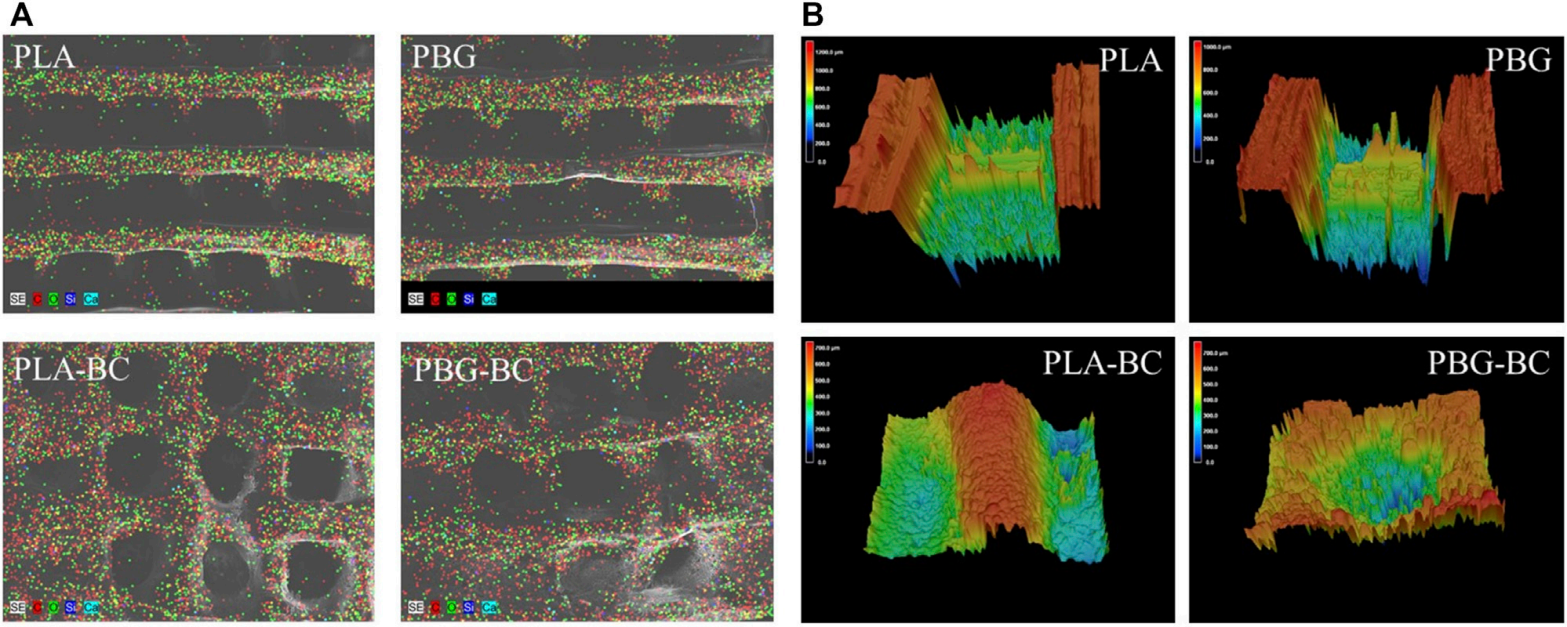
**(A)**. EDS of PLA, PBG, PLA-BC, and PBG-BC. **(B)** 3D confocal microscope of PLA, PBG, PLA-BC, and PBG-BC.

The surface was observed by SEM and EDS (Figures 8, 9). The surface of PLA was relatively smooth, while BG particles could be observed on the surface of PBG. The results of EDS confirmed the main elements of the skeleton structure of these scaffolds. BC was filled into the gaps of PLA-BC and PBG-BC, and the boundaries were obvious between BC and the composite scaffolds.

#### 3.2.3 Addition of BC did not change the surface properties of PBG

The 3D confocal microscope revealed the surfaces of the scaffolds (Figure 9). The surface roughness was significantly improved after the addition of BC. There was an obvious boundary between the surface of PBG and BC, indicating that BC did not change the original properties of the scaffolds.

#### 3.2.4 PBG-BC showed enough compressive strength

**FIGURE 10:**
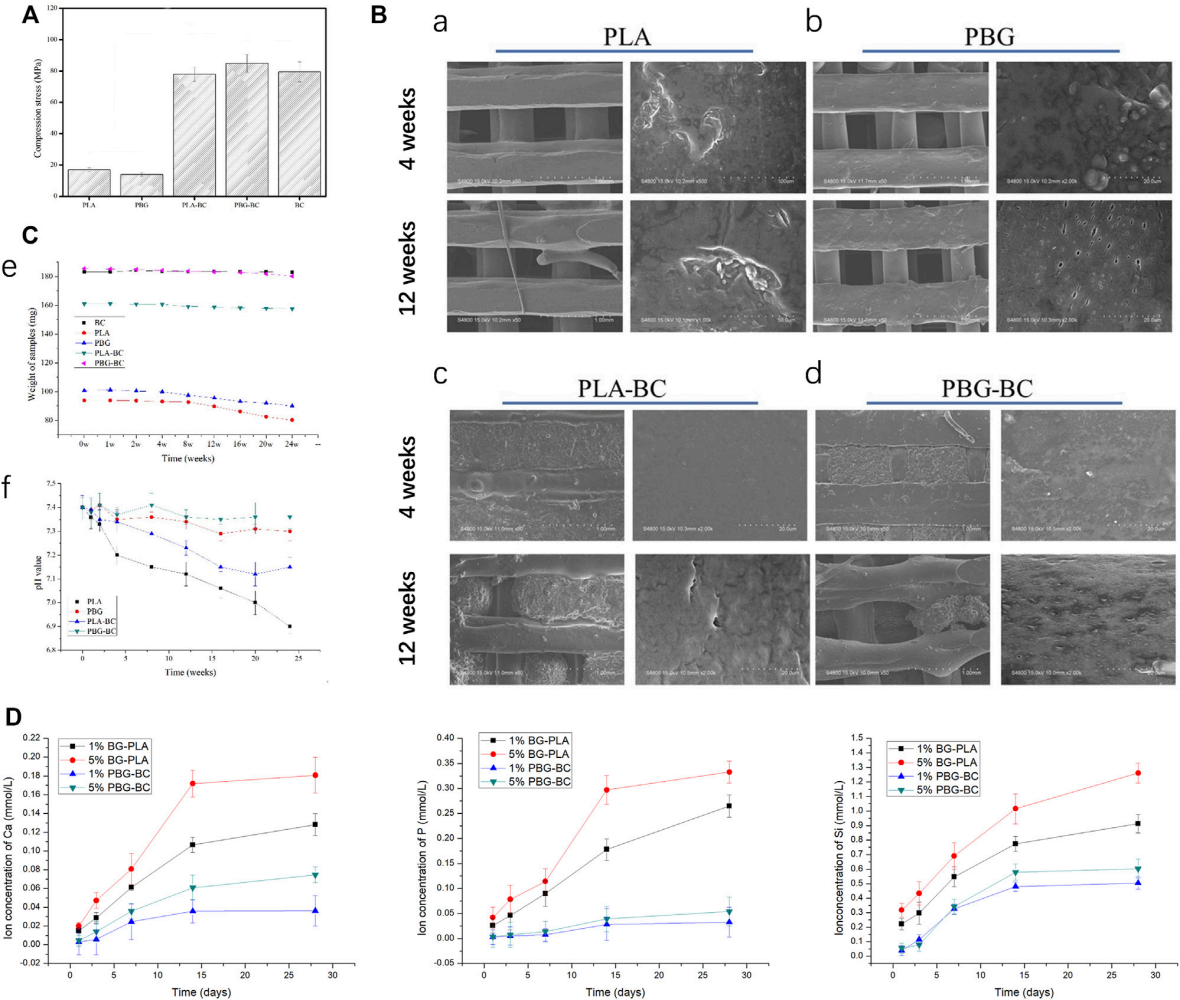
**(A)**. Compressive strength test of PLA, PBG, PLA-BC, and PBG-BC. **(B)**. SEM of PLA, PBG, PLA-BC, and PBG-BC soaked in Tris-HCl for 4 and 12 weeks. **(C)**. Sample weighing and PH test of PLA, PBG, PLA-BC, and PBG-BC soaked in Tris HCl for 24 weeks. **(D)**. Elemental concentration analysis of Ca, P, and Si.

The results of the compressive strength test showed that the compressive strength of PLA and PBG was only about 20 MPa (Figure 10). The values of PLA-BC and PBG-BC reached about 80 MPa, which was similar to that of PBG, indicating that the addition of BC significantly improved the compressive strength of the scaffolds.

#### 3.2.5 The weight and pH of PBG-BC changed slightly during degradation

Samples were soaked in Tris HCl for 24 weeks to analyze the degradation performance (Figure 10). After soaking for 4 and 12 weeks, SEM was performed to observe the surface of the samples. There was no obvious change on the surface of BC, while PLA and PBG were degraded to varying degrees. There were some defects on the surface of PLA, and small holes were observed on the surface of PBG. Similar degradation morphologies were found on the surfaces of PLA-BC and PBG-BC.

After soaking for 24 weeks, the sample weighing results showed that the weight gradually decreased in PLA and PBG groups, while the value changed slightly in PLA-BC and PBG-BC groups, indicating that the addition of BC influenced the degradation rate of scaffolds. The pH test results showed that the pH values gradually decreased in PLA and PLA-BC groups, while the values were relatively stable in PBG and PBG-BC groups, indicating that the degradation of BG could regulate the pH of the local microenvironment.

#### 3.2.6 BC had little influence on the cell adhesion of PBG-BC.

**FIGURE 11:**
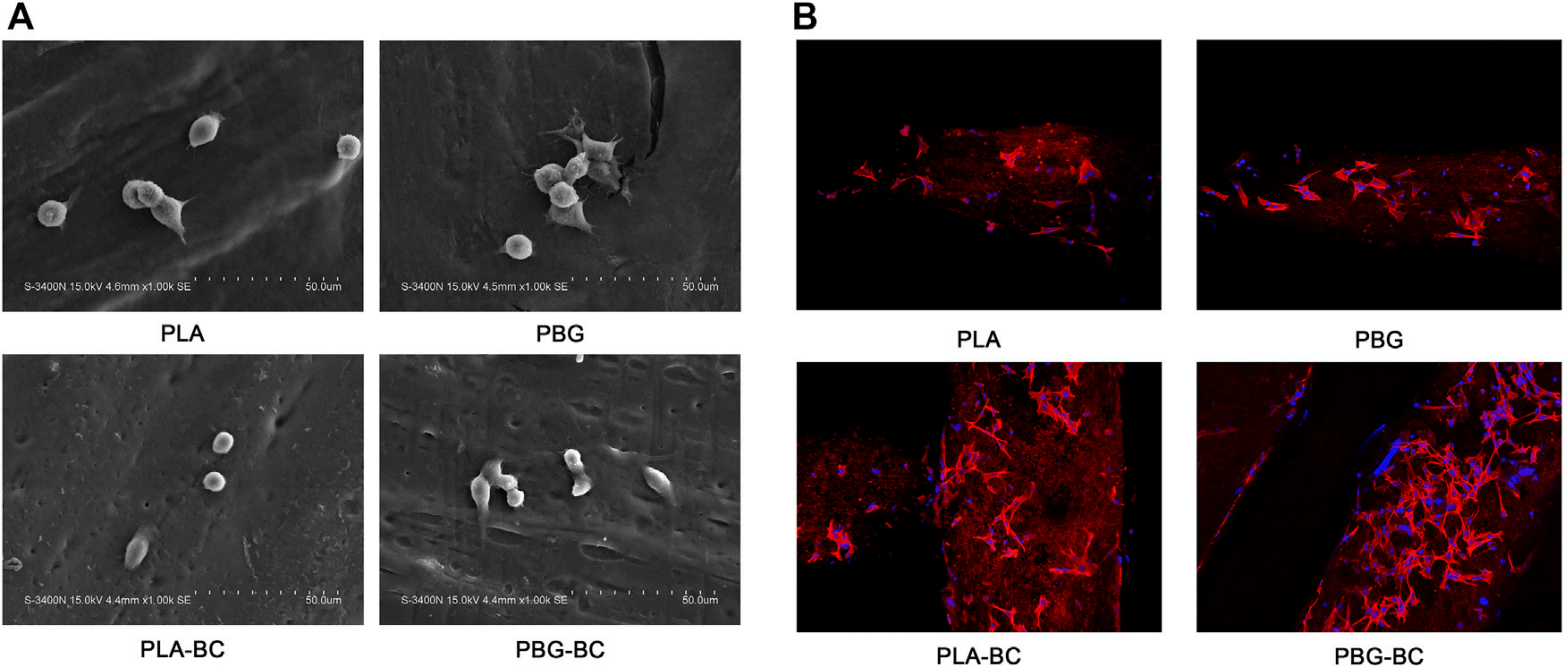
**(A)**. SEM of BMSCs cultured on the surface of PLA, PBG, PLA-BC, and PBG-BC. **(B)**. Laser confocal microscope of BMSCs cultured on the surface of PLA, PBG, PLA-BC, and PBG-BC.

The morphology and adhesion of BMSCs were observed by SEM and laser confocal microscope (Figure 11). The results of SEM showed that the cells had well-formed pseudopodia on the surface of PLA-BC and PBG-BC similar to that of PLA and PBG. The results of laser confocal microscopy showed that large amounts of cells were attached on the surface of PLA and PBG, except that the cells did not attach on the surface of BC.

#### 3.2.7 BC influenced little on the osteogenic induction ability of PBG

**FIGURE 12:**
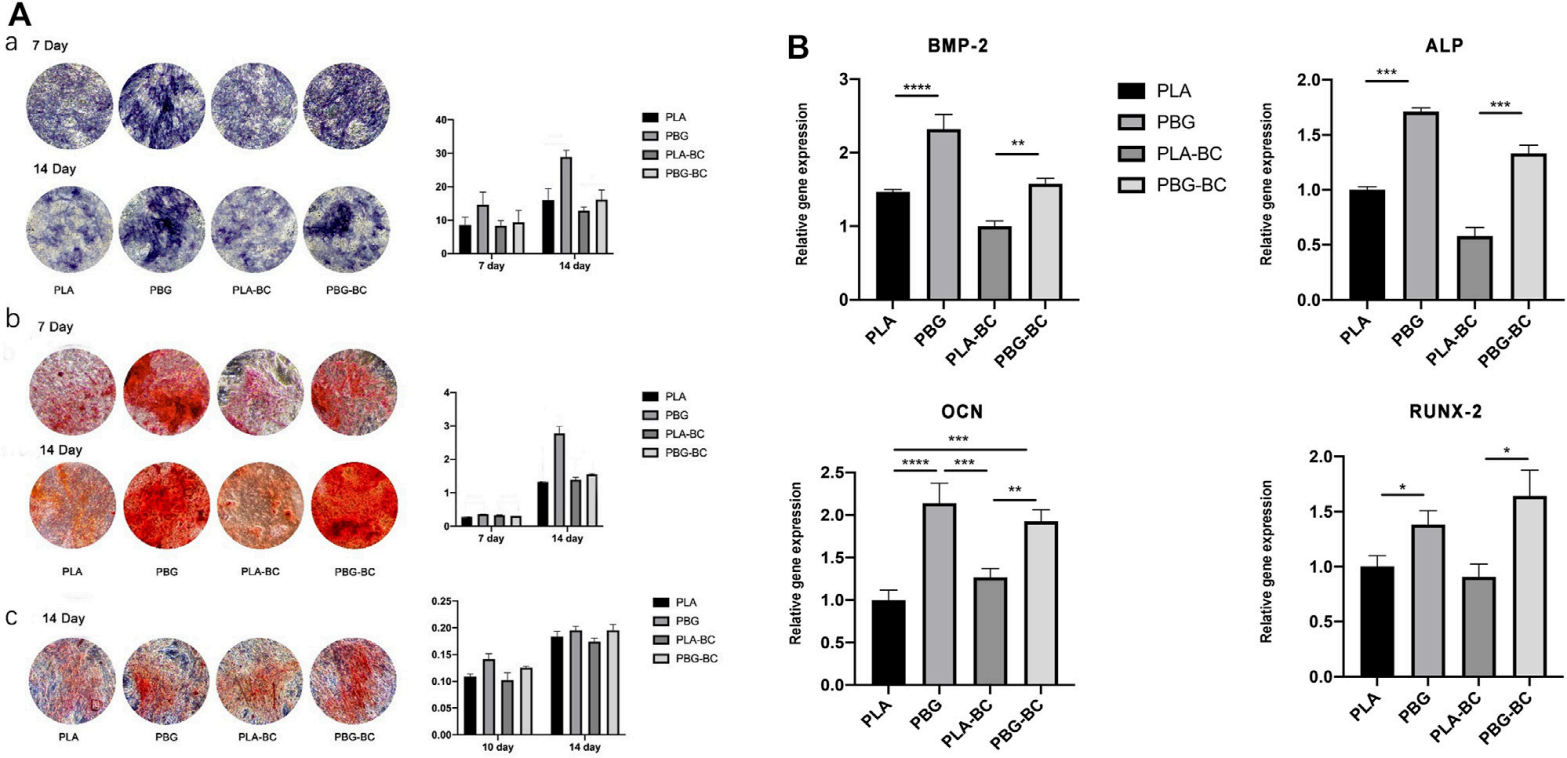
**(A)**. Evaluation of osteogenic differentiation of hBMSCs cultured with leaching solutions from PLA, PBG, PLA-BC, and PBG-BC. a: ALP staining and statistical analysis; b: Alizarin red staining and statistical analysis; c: Sirius red staining and statistical analysis. **(B)**. Relative expression of osteogenesis-related genes.

The osteogenic differentiation of BMSCs was evaluated by ALP staining, Alizarin red staining, and Sirius red staining (Figure 12). The results showed that PBG-BC could still induce osteogenic differentiation after adding BC. In the PBG-BC group, the staining area of calcium nodules was larger than that of PLA and PLA-BC, but smaller than PBG, indicating that BC affected the osteogenic ability of PBG.

RT-PCR was conducted to detect gene expressions of RUNX2, BMP-2, ALP, and OCN (Figure 12). The results showed that both PBG and PBG-BC significantly enhanced the relative expression of osteogenesis-related genes, and the addition of BC did not influence the promoting effect.

#### 3.2.8 Ca, P, and Si were cumulatively released from both PLA-BG and PBG-BC.

Elemental concentration analysis was conducted to detect releasing profiles of PBG and PBG-BC (Figure 10). Ca, P, and Si was cumulatively released in all the groups, and tended to plateau after 2 weeks. The releasing amounts of ions were higher in PLA-BG groups than that in PBG-BC groups, indicating that BC influenced ion release. The results also suggested that the concentration of BG also affected the releasing amounts of ions. The higher the concentration of BG, the more ions were released.

### 3.3 PBG-BC were firmly fixed in the femoral tunnel of New Zealand rabbits and induced new bone formation at the interface

#### 3.3.1 PBG-BC began to induce new bone formation after 4 weeks of implantation

**FIGURE 13:**
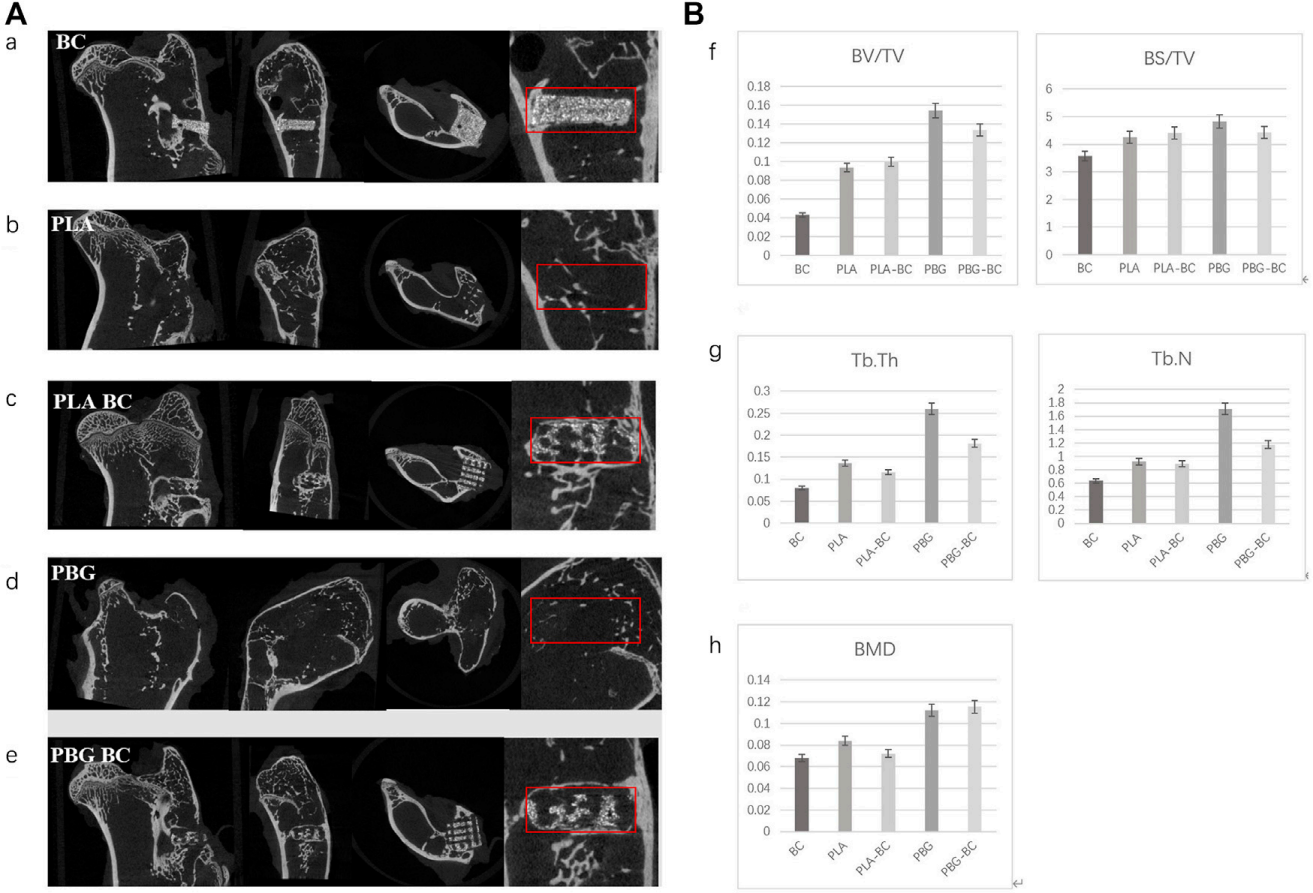
**(A)**. Coronal, sagittal, cross-sectional, and local enlarged images of micro-CT 4 weeks after sample implantation. a: BC, b: PLA, c: PLA-BC; d: PBG; e: PBG-BC. **(B)**. Statistical comparative analysis of local area (2 mm × 2 mm × 1 mm around the stent) of micro-CT 4 weeks after sample implantation. f bone volume/tissue volume (BV/TV), bone surface area/tissue volume (BS/TV); g: trabecular thickness (TB. Th), trabecular number (TB. N); h: bone mineral density (BMD). *: *p* < 0.05.

4 weeks after implantation, Micro-CT showed that PLA and PBG were partially degraded and replaced by a small amount of new bone (Figure 13). In PLA-BC and PBG-BC groups, the implants maintained a good cubic structure. A small amount of new bone was found in the gap, and new bone in the PBG-BC group was more dense. However, a clear boundary between bone and BC could still be seen in BC groups. The measurement of local areas showed that the groups containing BG (PBG and PBG-BC) were superior to pure PLA groups (PLA and PLA-BC) and BC group in terms of BV/TV, BS/TV, TB. Th, TB.N, and BMD (Figure 13). This result further proves that BG can induce osteogenesis and bone integration.

#### 3.3.2 PBG-BC was firmly fixed in the femoral tunnel and induced new bone constantly at the interface 12 weeks after implantation

**FIGURE 14:**
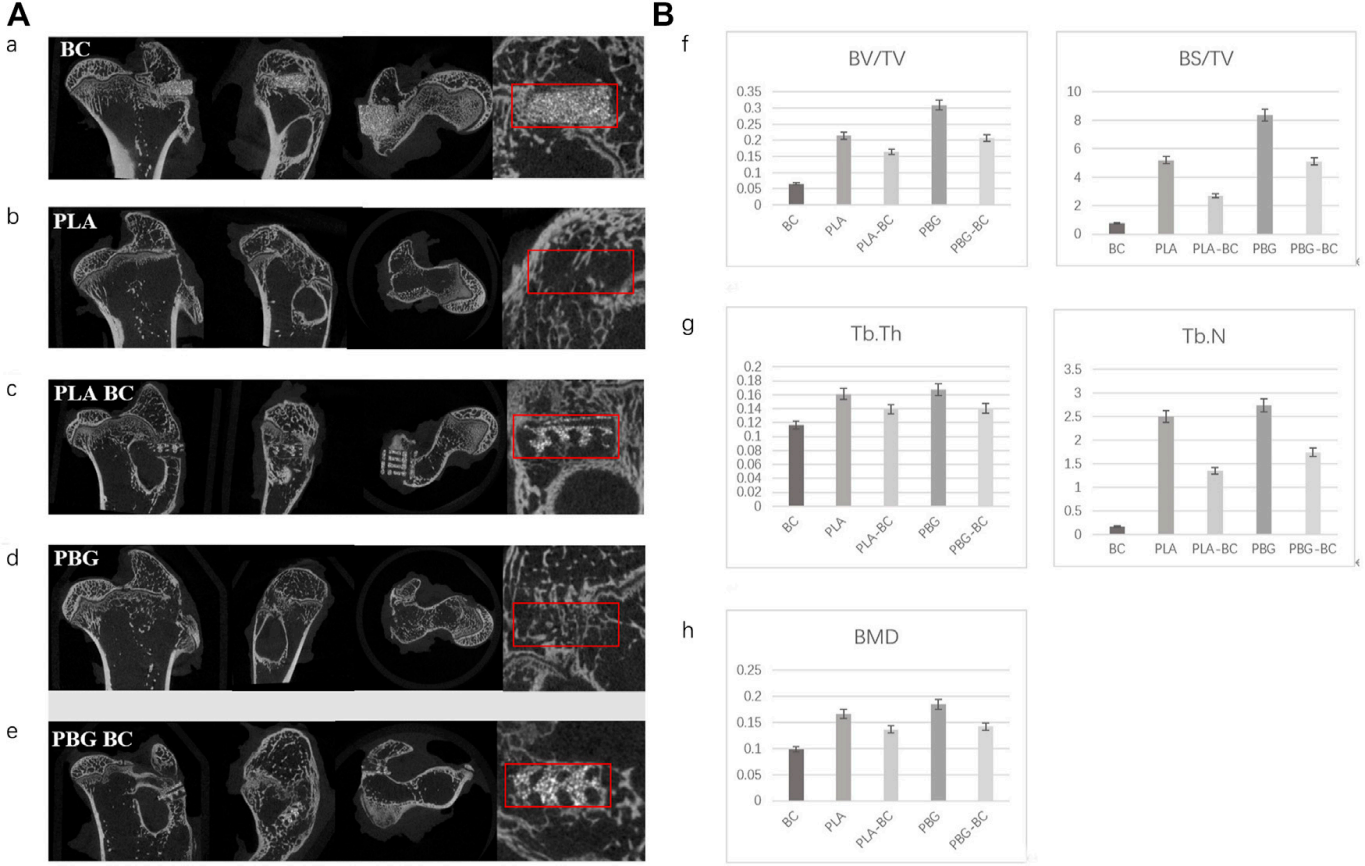
**(A)**. Coronal, sagittal, cross-sectional, and local enlarged images of micro-CT 12 weeks after sample implantation. a: BC, b: PLA, c: PLA-BC; d: PBG; and e: PBG-BC. **(B)**. Statistical comparative analysis of local area (2 mm × 2 mm ×1 mm around the stent) of micro-CT 12 weeks after sample implantation. f: BV/TV, BS/TV; g: TB. Th, TB.N; h: BMD. *: *p* < 0.05.

12 weeks after implantation, micro-CT showed that PLA and BG were further degraded, more bone tissues were found in the gap, new bone was more dense in PBG and PBG-BC groups, and PLA-BC and PBG-BC groups still maintained a good cube structure (Figure 14). A clear boundary was still found in the BC group. Local area measurement of BV/TV, BS/TV, TB. Th, TB.N, and BMD showed that PBG was better than PLA, and PBG-BC was better than PLA-BC, indicating that the addition of BG promoted new bone formation (Figure 14). The values also showed that PLA-BC was lower than that of PLA, and PBG-BC was lower than that of PBG, indicating that BC had some impact on new bone formation.

## 4 Discussion

An ideal filling system for bone defects in the weight-bearing area should provide strong initial mechanical support and bone regeneration ability. In this study, PLA and BG were prepared as frame structures by 3D printing technology, with PMMA as the filling structure. The composite has sufficient initial mechanical stability. BG can induce ion exchange during degradation, forming a local microenvironment conducive to bone regeneration, which significantly improves the long-term stability of the interface.

The frame structure is composed of PLA and BG. BG is silicate glass with good biocompatibility, degradability, and bioactivity. It is a commonly used bone tissue repair material clinically (Bento et al., 2021; Zheng et al., 2021; Daskalakis et al., 2022). BG has bone inducing effect. When BG is implanted into the body, it can exchange ions with extracellular fluid, and form a HA layer on its surface, to build a solid chemical bond with the bone surface (Daskalakis et al., 2022). In addition, different ions released by BG, such as silicon and calcium, can regulate osteoblast activity, promote extracellular matrix mineralization and accelerate new bone formation (Daskalakis et al., 2022). C Wu found that silicon ions at a certain concentration could stimulate cell proliferation, differentiation, and enhance bone mineralization and metabolism (Wu et al., 2007). P Valerio found that silicon ions could increase the cell viability of osteoblasts (Valerio et al., 2004). However, the mechanical strength of BG is too weak to be used for bone tissue repair alone. To provide mechanical strength, PLA was selected as the loading scaffold for BG. PLA is known as a degradable polymer, the degradation product is nontoxic and has good mechanical properties (Kaseem et al., 2021). The results of the study showed that the composite of the two materials not only improved the mechanical properties and elastic modulus but also met the requirements of bone induction.

The results of the degradation performance test showed that the degradation rate of pure PLA was about 20%, while the degradation rate of PLA-BG rose to 25%, indicating that the addition of BG significantly improved the degradation rate. This phenomenon may result from the interaction of degradation products of PLA and BG. After the degradation of pure PLA, the local internal environment became acidic, which resulted in the decrease in the degradation rate (Vaid et al., 2021). The degradation products of BG are alkaline (Daskalakis et al., 2022), which has an acid-base neutralization effect on the local internal environment, maintains the steady-state of the internal environment, and is conducive to the continuous occurrence of degradation. After adding bone cement, the degradation rate of PBG-BC decreased significantly to 10%, which may be due to the significant reduction of the direct contact area between the material and the surrounding tissue. The results of the ion releasing test showed that the ion concentration released by PBG-BC was significantly lower than that of PLA-BG. In addition, bone cement itself is of high quality and is barely degradable, which also may affect the degradation rate.

Cell adhesion rate and protein adsorption of the material have a significant impact on cell activity (Li et al., 2021). Pure PLA produced an acidic degradation environment, which was not conducive to cell adhesion and proliferation, so the cell adhesion and proliferation rate were low. It is reported that the pure BG powders usually resulted in a high pH value in the implanted site (Zhu et al., 2020). In our study, the combination of BG and PLA made the pH value stable within the normal physiological range, and the cell adhesion and proliferation rate were significantly higher than that of pure PLA. BG degradation products such as Ca, P, and Si are also conducive to cell proliferation and adhesion (Daskalakis et al., 2022). As for the filling structure in the system, bone cement is bioinert material, the addition of bone cement has little impact on the local environment and cell activity.

Once the scaffolds are implanted into the defect site, foreign body responses occur because of the inflammatory reactions of the host to the implants, which could delay the repairing process (Williams, 2008). An improved understanding suggested that the biomaterial-immune system interaction plays a far more important role in tissue regeneration (Loi et al., 2016; Vishwakarma et al., 2016). The interactions between implants and macrophages have an important impact on interface inflammatory response and tissue reconstruction (Liu et al., 2021). Studies have shown that macrophages can support bone formation by recruiting mesenchymal stem cells (MSC) to the defect site (Freytes et al., 2013; Wynn and Vannella, 2016). Macrophages can also enhance the angiogenesis of endothelial cells (Dong et al., 2017). It is known that the change in the microenvironment can polarize macrophages in either direction of M1 or M2 (Ma et al., 2021). M1 macrophages can secrete pro-inflammatory cytokines while M2 macrophages take part in tissue remodeling and resolution of inflammation (Loi et al., 2016). It was reported that macrophages of the M2 phenotype could enhance the expression of bone morphogenetic protein-2 (BMP-2) in macrophages, which regulated osteogenic differentiation of BMSC (Chen et al., 2014). The results of this study showed that pure PLA promoted M1 polarization of macrophages, which may be due to the acidic environment produced by PLA, and some particles dissolved by PLA have a pro-inflammatory effect. PBG induced M2 polarization of macrophages, which may be due to the degradation microenvironment caused by BG. Studies have shown that ionic products of BG could activate macrophages toward the M2 phenotype and stimulate macrophages to secrete anti-inflammatory growth factors (Dong et al., 2017).

The composites were prepared by 3D-based FDM, which is a physical process without additives in the preparation process (Grivet-Brancot et al., 2022). The method can ensure the original characteristics of the material. The preparation time is short and can be customized according to different anatomical information. The high porosity of biomaterials is conducive to the growth of cells and blood vessels. PLA-BG prepared in this study has a completely interconnected channel network, which is conducive to bone growth.

The *in vivo* experiments revealed the structural relationship and bone integration between scaffolds and surrounding bone tissue. 4 weeks after implantation, the composite scaffold with BG, whether or not containing bone cement, had better bone integration, indicating that BG could promote new bone formation in the early stage. The composite scaffold containing bone cement maintained a good frame structure, but no obvious osteogenic reaction was found in the simple bone cement group, indicating that bone cement can only provide the initial stability of the scaffold. After 12 weeks of implantation, with the continuous degradation of PBG, a large number of new bones filled the space after degradation, while new bone formation in the simple bone cement group was still not obvious, indicating that PBG can continuously promote osteogenesis. PBG-BC maintained a good cubic structure, but the formation of new bone was worse than PBG, which may be due to the space occupied by bone cement could not form new bone. Although PBG had the best bone ingrowth performance, the scaffold may fail in the early stage without the support provided by bone cement.

## 5 Conclusion

PBG-BC could provide immediate and stable fixation for a bone defect in a load-bearing area. It also had the characteristics of continuous osteogenic induction. The continuous degradation of PBG provided space for bone growth. During the degradation process, the release of various elements promoted osteogenic metabolism, regulated the local microenvironment, induced M2 polarization of macrophages, inhibited local inflammatory response, and provided long-term stability via bone integration.

## Data Availability

The original contributions presented in the study are included in the article/Supplementary Material, further inquiries can be directed to the corresponding author.
